# Latrine access and utilization among people with limited mobility: A cross sectional study

**DOI:** 10.1186/s13690-016-0120-5

**Published:** 2016-03-01

**Authors:** Berhanu Asfaw, Muluken Azage, Gebremedhin Berhe Gebregergs

**Affiliations:** Organization for Rehabilitation and Development in Amhara (ORDA), MDG - Urban Sanitation Project, P.O.Box 132, Bahir Dar, Ethiopia; School of Public Health, College of Medicine and Health Sciences, Bahir Dar University, P.O.Box 79, Bahir Dar, Ethiopia

**Keywords:** Latrine access, Latrine utilization, People with limited mobility, Disability

## Abstract

**Background:**

Latrine access is one of the challenges faced by people with physical disabilities that limit their mobility (PPDs) in their home and working environments. Latrines should be designed, built and located such that they are easily accessible and utilizable by PPDs. Therefore, the aim of this study was to determine latrine access and utilization, and explore the challenges in latrine use among PPDs in Bahir Dar city, northwest Ethiopia.

**Methods:**

A cross-sectional study design was conducted from July 15 to August 15, 2014. Data were collected using a structured and pre-tested questionnaire, and focus group discussions. Four hundred nineteen participants were included using a systematic random sampling technique. SPSS version 20 was used for data entry and analysis. Binary logistic regression was used to identify factors associated with latrine utilization. Qualitative data were analyzed using themes.

**Results:**

Of 419 participants, 142 (33.9 %) had access to latrines and 173 (41.3 %) had satisfactory latrine utilization. Family support while using latrine (AOR = 4.7, 95 % CI (2.7, 8.3), latrine accessibility (AOR = 2.1, 95 % CI (1.2, 3.7) and past latrine modification (AOR = 3.1, 95 % CI (1.8, 5.4) were factors associated with latrine utilization. Presence of steps at the latrine entrance, privacy while using latrine, absence of handrails, unavailability of family support, narrower latrine door, distant latrine, unclean floor of the latrine and elevated foot rests were challenges mentioned by PPDs.

**Conclusions:**

Latrine access and utilization were low among PPDs. Family members should encourage and support PPDs when they need to use latrine, designing accessible latrines, modifying existing latrines to accommodate PPDs are the areas of interventions to increase latrine accessibility and utilization among PPDs.

## Introduction

In developed as well as developing countries, people with physical disabilities that limit their mobility (PPDs) face difficulties in their physical environments [1–4]. PPDs generally have poorer health, lower educational achievements, fewer economic opportunities and higher rates of poverty than physically-abled or fully-mobile people, due to lack of access to a range of services [1]. Lack of access to water and sanitation services that are enjoyed and utilized by those without mobility challenges (either temporary or permanent) is a denial of human rights [1, 3].

Sanitation is one of the services that is often inaccessible for PPDs in their homes and communities [1]. Studies have documented that latrines or toilets in public and institutional settings, such as transportation centers, markets, schools and medical facilities are not accessible to PPDs [5–8]. Even at home, accessibility of latrines for PPDs is very limited, affecting their quality of life [9–11]. As with the non-disabled population, correct and consistent use of existing latrines is a challenge. Even when there are disabled-accessible latrines, use of those latrines by PPDs was still low [1]. Lack of knowledge about accessibility issues for PPDs was mentioned as a factor that affects the provision of appropriate latrines or toilets for PPDs [1], and behavior change materials and messages may need to be modified to support adoption of improved sanitation behaviors by PPDs.

Access and regular use of sanitation facilities by PPDs in homes and workplaces has an important role in reducing their risk of developing diseases associated with poor sanitation, as well as in promoting their health and prevention of other diseases associated with disability, like under-nutrition [12–14]. Lack of sanitation facilities compels people to practice open defecation which increases the risk of disease transmission for the whole community [15, 16]. In addition, women with physical disabilities are especially vulnerable to assault when they lack access to a sanitation facility, since they often take advantage of the darkness to relieve themselves, and they cannot outrun an attacker [1].

In Ethiopia, there has been rapid progress on construction of sanitation facilities in all parts of the country since 2003, through the introduction of the Health Extension Program by the Ministry of Health. The availability of improved and shared latrine facilities at household level had increased from 8 % in 2000 to 47 % in 2012 [17]. The second National Health Sector Transformation Plan of Ethiopia set a goal of 82 % latrine coverage to improve sanitation and hygiene across the country by 2019 [18].. However, most non-governmental organizations and government implementers have not addressed disability-focused latrine access and utilization interventions. In addition, the Millennium Development Goals (MDGs) which had generated concern among the global community did not specifically consider the accessibility of latrines for PPDs [19]. Therefore, the aim of this study was to assess latrine access and utilization; identify factors associated with utilization; and explore challenges related to latrine use, among PPDs in Bahir Dar city, Amhara State, Ethiopia.

## Methods and materials

### Study design, setting and source population

The study used a community-based cross-sectional study design with both quantitative and qualitative methods. The study was conducted from July 15 to August 15, 2014 in Bahir Dar city, the capital of Amhara National Regional State in northwest Ethiopia. The city has a total population of 284,020 (47.5 % male) in nine urban sub-cities [20]. According to the City Labor and Social Affairs Office report (2014), a total of 2,245 people with disabilities (PDs) lived in the city, of which 1,637 (73 %) were people with physical disabilities [21]. Of all the people with physical disabilities in Bahir Dar, only 1,421 (87 %) lived in households with a latrine [21].

### Study population and variables

The selection criteria included all people with physical disabilities living in the study area, age greater than or equal to 15 years, and who had lived at least six months in a household with some type of a latrine. The dependent variables were access to PPD-accessible latrines (as defined for this study) and utilization, whereas the independent variables to explore latrine utilization by those PPDs with access included socio-demographic characteristics, latrine-related and environmental factors.

### Sample size and techniques

A single population proportion formula was used to calculate sample size using Epi Info version 3.5.3, and based on the following assumption: 50 % proportion of latrine access among PPDs (since there was no previous study and it gave adequate minimum sample size). The sample size selection included a 5 % of margin of error, 95 % confidence level, and 10 % non-response rate. The final sample size was 422. Participants were selected using a systematic random sampling technique using list of households that had at least one person with limited mobility of the City Labor and Social Affairs Office as sampling frame. If there were more than one person with limited mobility in a household, lottery method was used to select one participant.

### Data collection techniques

Nine enumerators (four men and five women) and three supervisors were recruited for the study, and one-day training was provided for all of the staff on proper data collection methods and how to facilitate focus group discussions. A structured questionnaire in the local Amharic language was developed and pre-tested. The final version was used to collect the quantitative data, including socio-demographic characteristics of PPDs, latrine-related and environmental factors, and latrine utilization. A focus group guide was used to collect the qualitative data, which covered latrine access and utilization, and challenges for latrine utilization. Four focus group discussions were conducted; two with men participants only and two with women only, based on the RATS guidelines [22]. Students, individuals who had no job at the time of study, private and government employees were focus group discussants. Focus group discussants were selected from different kebeles of the City Administration and were not a subset of the participants in the quantitative survey. In each focus group discussion, there was one facilitator and one note taker, and the discussion was also recorded on an audio tape recorder.

### Data analysis

Data were entered, cleaned and analyzed in SPSS version 20 software. Descriptive statistics such as mean and proportion were used to describe the data. Bivariate and multivariable logistic regression analyses were performed to identify factors associated with latrine utilization. In bivariate logistic regression, variables whose p-value is ≤ 0.2 were retained for multivariable logistic regression analysis. Adjusted odds ratios (AORs) with 95 % CIs were calculated to identify predictors of latrine utilization. The audio data from the focus group discussions were transcribed into text, and the qualitative data were analyzed manually using a content thematic approach, following a framework suggested by the researchers [24].

### Operational definitions

1. People with Physical disabilities is persons who for whatever reason:
  - Cannot walk, and may use a wheelchair, trolley, or other mobility device.
  - Can walk with difficulty, and need support from e.g. crutches, handrail, or person to lean on.
  - Can walk, but experience other physical weakness or lack of coordination including weakness in legs [23].
2. Functional latrine: a latrine that provides services for household members at the time of data collection
3. Satisfactory latrine utilization includes ALL of the following: PPDs live in households with functional latrines and either PPDs self-report using it by themselves or with family support on a regular basis; there are no other means of defecation (potty/bowl) present.
4. Accessible latrine for PPDs includes ALL of the following: PPDs having functional latrine that is sufficiently close to their dwellings (≤6 m); without any steps; at least 1 m wide clear path that accommodates any mobility aids (crutches, wheelchair, etc.); a minimum 1 m door width and 1 m square latrine room space inside; and presence of handrails [24].
5. Past latrine modification is used for those latrines that have been previously constructed inaccessibly, but they are accommodated or are being made suitable for use by people with disabilities, for example, building wheelchair ramps.

### Ethical Considerations

The study was approved by Ethical Review Committee of College of Medicine and Health Sciences, Bahir Dar University, after reviewing the research protocol including the ethical procedures. A letter of permission was obtained from the Amhara National Regional Health Bureau. Data were collected using interviewer administered questionnaire and observational checklist. Informed oral consent was obtained from each participant and parents (for those participants having age < 18 years) after explaining the purpose of the study. Participants were assured of confidentiality with regard to all information acquired. In addition, during the interviews, each participant was informed that he or she was free to withdraw at any time from the study.

## Results

### Socio-demographic characteristics

A total of 419 PPDs participated in the study. The majority of the participants, 229 (55 %), were males, and in the age range of 20–24 years (29 %). The mean age of participants was 29.9 ± 12.5 (SD) years. Of the total participants, 188 (45 %) were unable to read and write. By occupation, 169 (40 %) were merchants and 85 (20 %) were students. With regard to the income status of the participants, 132 (32 %) had a monthly income of less than or equal to 200 Birr (9.65 US dollar) (Table 1).

**Table 1:** Socio-demographic characteristics of the respondents in Bahir Dar, Northwest Ethiopia, 2014 (*n* = 419)

Variable	Category	Frequency	Percent
Age	15–19	66	15.8
	20–24	123	29.4
	25–29	79	18.9
	30–34	39	9.3
	35–39	32	7.6
	≥40	80	19.1
Sex	Male	229	54.7
	Female	190	45.3
Marital Status	Single	199	47.5
	Married	138	32.9
	Divorced	61	14.6
	Widowed	21	5.0
Educational Status	Unable to read/write	188	44.9
	Read and write including grade 1–8	153	36.5
	Grade 9–12	68	16.2
	Certificate and above	10	2.4
Occupational Status	Employed(any)	42	10.0
	Merchant/IGA activities	208	49.6
	House wife	88	21.0
	Daily laborer	57	13.6
	Jobless	24	5.7
Monthly Income (Ethiopian currency)	≤200 birr	132	31.5
	201–300 birr	119	28.4
	301–400 birr	88	21.0
	≥401 birr	80	19.1
Membership to disability association	Yes	212	49.4
	No	207	50.6

### Latrine access and utilization, latrine related and environmental factors

Only 142 (34 %) participants had PPD accessible latrine access according to the definition used in this study. One hundred and seventy three participants (41 %) reported that they always used the latrine in their household (whether it met the definition of PPD accessible latrine or not), 144 (34 %) reported that they used the latrine rarely and 102 (24 %) reported that they most often used the latrine. When asked about other places used for urination and/or defecation, 154 (62 %) reported that they used an open field, 61 (25 %) used a potty/bowl and 22 (9 %) used nearby bushes. Of all participants, 173 (41 %) had satisfactory latrine utilization (Table 2).

**Table 2:** Level of latrine utilization, latrine related and environmental factors among persons with physical disabilities in Bahir Dar city, 2014

Variable	Category	Frequency	Percent
Frequency of latrine utilization	Always	173	41.3
	Mostly	102	24.3
	Rarely	144	34.4
Means of defecation other than latrine (*n* = 246)	Open field	153	62.3
	Potty/bowl	61	24.7
	Nearby bushes	22	8.9
	Drainage ditches	8	3.2
	Others**	2	0.8
Walkway to latrine allows mobility assistance device (wheelchair, crutches, etc.)	Yes	208	49.6
	No	211	50.4
Level of latrine utilization	Satisfactory	173	41.3
	Unsatisfactory	246	58.7
Distance of latrine from household	<= 6 metaers	163	38.9
	>6 m	256	61.1
Latrine accessibility	Accessible	142	33.9
	Inaccessible	277	66.1
Family support while using latrine	Yes	239	57.0
	No	180	43.0
Type of latrine	Traditional dry pit latrine	315	75.2
	Ventilated improved pit latrine	55	13.1
	Pour flush latrine	41	9.8
	Others**	8	1.9
Availability of Hand washing facility	Yes	187	44.6
	No	232	55.4
Privacy while using latrine	Yes	240	57.3
	No	179	42.7
Falling history inside latrine room	Yes	191	45.6
	No	228	54.4
Injury history when falling in latrine room (*n* = 191)	Yes	115	60.2
	No	76	39.8
Latrine modification in the past (at least one trial)	Yes	206	49.2
	No	213	50.8

Means of defecation other than latrine- Others**- Plastic containment (flying toilets)

Type of latrine- Others** - (Modified bucket type latrines)

Two hundred eight (50 %) participants had wide latrine walkways allowing the mobility assistive devices to pass smoothly and the rest, 211 (50 %), had grasses, bushes and other barriers blocking the access path to the latrine. One hundred ninety-one (46 %) participants had at least one experience of falling in the latrine room of which 115 (60 %) were injured during the fall. Two hundred six (49 %) latrines were modified in the past to make accessible for PPDs. Only 163 (39 %) latrines were found at recommended distance. Most of the latrines, 277 (66 %) were inaccessible for PPDs. Only 70 (17 %) latrines had supporting handrail (Table 2).

### Reasons for irregular use of the latrines

**Fig. 1:**
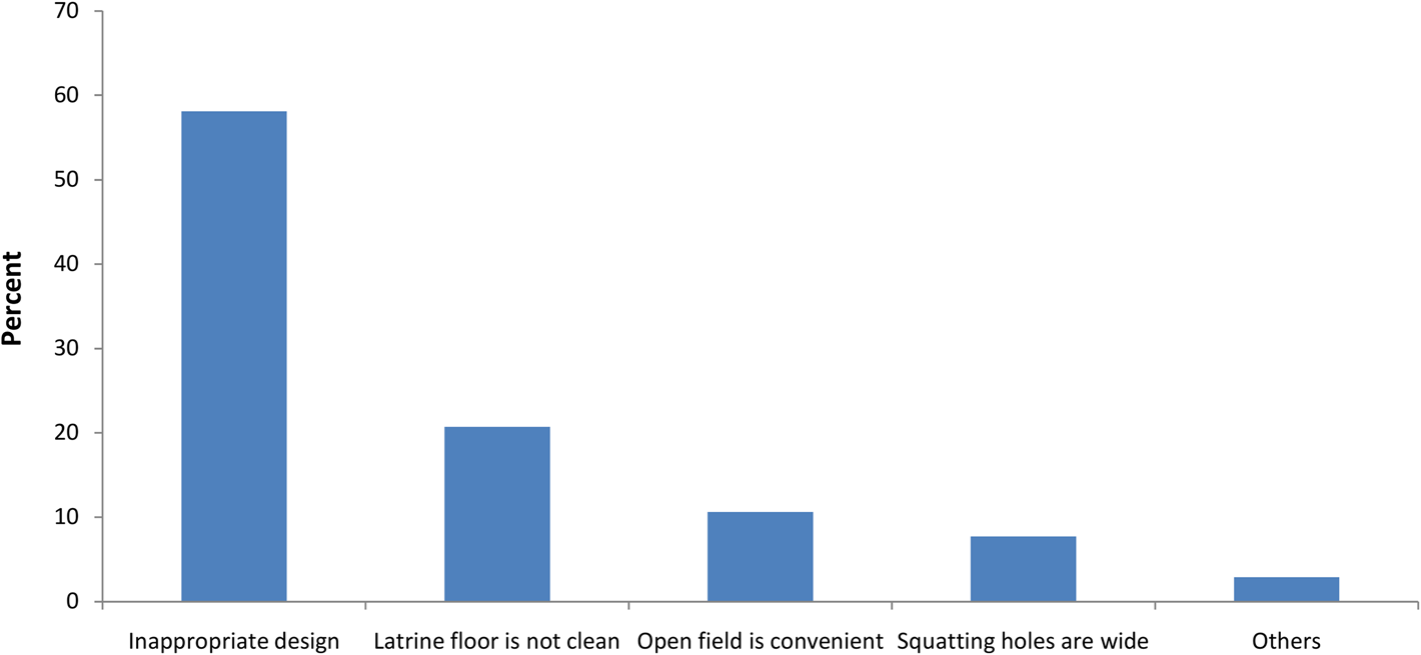
Reason for irregular use of latrine among persons with physical disabilities in Bahir Dar city, 2014

In the survey, inappropriate design (58 %), ‘unclean latrine floor’ (21 %), ‘open field is convenient’ (11 %), and ‘too wide squatting holes’ (8 %) were reasons mentioned by PPDs for not regularly using the household latrines (Fig. 1).

### Factors associated with latrine utilization among PPDs

In bivariate logistic regression analysis, age, income, privacy, family support while using latrines, latrine modification in the past and latrine accessibility were factors associated with latrine utilization with a p-value less than 0.2. These variables were further included in the multivariable logistic regression analysis.

In multivariable logistic regression, family support, having a PPD accessible latrine, and past latrine modification had statistically significant associations with latrine utilization. PPDs that have a family support while using latrine were 4.7 times more likely to have satisfactory latrine utilization (AOR = 4.7, 95 % CI (2.7, 8.3). Those PPDs who had accessible latrines were 2 times more likely to have satisfactory latrine utilization than those with inaccessible latrines (AOR = 2.1, 95 % CI (1.2, 3.7). PPDs who made a latrine modification in the past were 3 times more likely to use latrine as compared to PPDs with no past latrine modification (AOR = 3.1, 95 % CI (1.8, 5.4) (Table 3).

**Table 3:** Binary and Multivariable Logistic Regression on factors associated with latrine utilization among PPDs in Bahir Dar, 2014

Variables	Category	Latrine utilization		COR (95 % CI)	AOR (95 % CI)
		Satisfactory (%)	Unsatisfactory (%)		
Sex	Male	103 (45.0)	126 (55.0)	1.4 (1.0–2.1)	
	Female	70 (36.8)	120 (63.2)	1.0	
Age	15–19	18 (27.3)	48 (72.7)	0.7 (0.3–1.4)	
	20–24	61 (49.6)	62 (50.4)	1.8 (1.0–3.3)	
	25–29	37 (46.8)	42 (53.2)	1.6 (0.9–3.1)	
	30–34	16 (41.0)	23 (59.0)	1.3 (0.6–2.8)	
	35–39	13 (40.6)	19 (59.4)	1.3 (0.6–3.0)	
	>40	28 (35.0)	52 (65)	1.0	
Educational status	Unable to read/write	74 (39.4)	114 (60.6)	1.00	
	Read and write including grade 1–8	61 (39.9)	92 (60.1)	1.0 (0.7–1.6)	
	Grade 9–12 (high school)	31 (45.6)	37 (54.4)	1.3 (0.7–2.3)	
	Certificate and above	7 (70.0)	3 (30.0)	3.6 (1.0–14.3)	
Occupation	Employed (any)	18 (42.9)	24 (57.1)	1.8 (0.6–5.3)	
	Merchant/IGA activities	95 (45.7)	113 (54.3)	2.0 (0.8–5.1)	
	House wife	38 (43.2)	50 (56.8)	1.8 (0.7–4.9)	
	Daily laborer	15 (26.3)	42 (73.7)	0.9 (0.3–2.5)	
	Jobless	7 (29.2)	17 (70.8)	1.0	
Membership to disability association	Yes	21 (9.9)	191 (90.1)	0.04 (0.02–0.1)	
	No	152 (73.4)	55 (26.6)	1.0	
Privacy	Yes	112 (46.7)	128 (53.3)	1.7 (1.1–2.5)	
	No	61 (34.1)	118 (65.9)	1.0	
Monthly income (Ethiopian Birr)	≤200 birr	54 (34.1)	87 (63.9)	1.0	
	201–300 birr	50 (42.0)	69 (58.0)	1.4 (0.8–2.3)	
	301–400 birr	36 (41.0)	52 (59.0)	1.3 (0.8–2.3)	
	≥401 birr	42 (52.5)	38 (47.5)	2.1 (1.2–3.8)	
Family support	Yes	139 (58.2)	100 (41.8)	5.9 (3.8, 9.4)	4.7 (2.7, 8.3)
	No	34 (18.9)	146 (81.1)	1.0	1.0
Past latrine modification	Yes	116 (56.3)	90 (43.4)	3.5 (2.3,5.3)	3.1 (1.8, 5.4)
	No	57 (26.8)	156 (73.2)	1.0	1.0
Latrine accessibility	Accessible	76 (53.5)	66 (46.5)	2.14 (1.4, 3.2)	2.1 (1.2, 3.7)
	Inaccessible	97 (35.0)	180 (65.0)	1.0	1.0

### Qualitative result

Seven themes were identified during the exploration of challenges for latrine utilization from the focus group discussions.

### Presence of steps at latrine entrance (Theme 1)

The presence of steps at the entrance of latrine door was raised as a big challenge of PPDs for not using latrine regularly. An 18 year old woman said, “*I sometimes intentionally slept hungry in order not to go latrine at night, since the latrine is not accessible.* A 22 year old male noted that “*when sometimes I fall on latrine steps, my family usually says “Who forced you to use latrine?” and further suggest that it would be fine if I used open field.”*

### Privacy while using latrine (Theme 2)

A 21 year old female explained that “*…. as a female, we need extreme privacy in using the latrine”.* A 29 year old man who used a crutch said “*Privacy is a significant issue. Take me as an example; I would rather use nearby forests even if it is harder than being seen by others while defecating.*”

### Availability of supporting handrails (Theme 3)

Availability of supporting handrails was identified as a barrier to use of latrines that were not PPD accessible. For instance, a 38 year old woman said, “*I mostly use the open field because the toilet floor is slippery and there is nothing to hold on the wall.”*

### Family support (Theme 4)

Family support enabled latrine use for PPDs. A 34 year old man said “*Above all things, for us, family support is ideal. Even if latrine has design problems, with family support we can always use it. My elder son helps me always while using the latrine.*”

### Narrow latrine door (Theme 5)

The width of the door to the latrine can cause challenges for access. A 33 year old male explained that “*I myself use a wheelchair, and I can’t get into the latrine with the wheelchair because the latrine door is narrow and old, and its nails are left sticking out of the wood.*”

### Distance of latrine from household (Theme 6)

When discussing proximity of the latrine, a 23 year old female student, said that “*The lucky ones have their latrine at home*”. Another 25 year old woman who used two crutches said *“There is no question at all about the necessity of latrine. But I always struggle to use it. The struggle starts from the location where it was built. My family latrine is at the corner of our compound and the path to the latrine is covered with bushes and grass.*”

### Unclean floor and elevated squatting foot rests (Theme 7)

A 22 year old woman said that “*The main reason for me for not using the latrine regularly is the cleanliness of the floor, it is disgusting and offensive. Some family members, especially young children, are urinating and defecating outside the squatting hole and all day long the latrine floor stays unclean and it’s uncomfortable for me to use.*”

## Discussion

Although presence of a household latrine was one of the selection criteria for the study, latrine access for PPDs (using the definition in this study) was only 34 % of the participants, showing that most available latrines are not accessible for PPDs. The reasons might be due to the little attention given by government and non-governmental organizations to support construction of accessible latrines for PPDs, low awareness of PPD needs in latrine design in the communities, and/or lack of financing by households with PPDs. The World Health Organization (WHO) suggests that awareness-raising and challenging negative attitudes are the first steps towards having accessible latrines for PPDs [1].

The latrine utilization rate was 41 %, which is in line with the results of a study carried out in Mali on which 42 % of the PPDs regularly used latrines [25]. However, it is lower than the second national health sector transformation plan of Ethiopia: to reach latrine coverage 82 % by the end of 2019 [18]. The low latrine utilization in the current study might be due to low level of awareness and accessibility of latrine for PPDs. The National Hygiene and Sanitation Strategic Action Plan clearly set a strategy of ensuring proper construction and hygienic latrines which could be used by all members including PPDs [26].

Family support in using latrines, latrine accessibility and past latrine modification were factors associated with latrine utilization among people with physical disabilities in the study area. PPDs that had family support were 4.7 times more likely to have satisfactory latrine utilization. This is in line with finding from Mali where family support was significantly associated with latrine [27]. Moreover, during the focus group discussions, family support was a principal factor to develop courage and confidence to regularly utilize a latrine.

PPDs with accessible latrines were two times more likely to have satisfactory latrine utilization as compared to their counterparts. The focus group discussion participants outlined the necessity of step-free latrines to support accessibility for those with mobility challenges.

PPDs whose household latrine has been modified in the past were three times more likely to use the latrine as compared to PPDs with no past latrine modification of the standard latrine design. The finding was supported in a focus group discussion, where a latrine was modified with a wider doorway and rope pull to make the latrine more comfortable for a woman using double crutches. Whenever existing latrines are modified to accommodate physically disabled family members, the chance of utilization increases as reported during a disability-focused program by Plan International Kenya in Kilifi, Kenya. The Kenyan study was a good example where disabled people had modified latrines to suit their situation, and the modifications included raised toilet seats, which allow users to sit comfortably, and use of two raised blocks on either side of the drop hole to avoid squatting [28].

This study has some limitations. The cross sectional study design limits the ability to establish cause and effect relationships with the variables and the outcomes. In addition, only mobility issues were included, not other types of disabilities like visual and hearing disabilities.

## Conclusions and recommendations

PPD accessible latrines and latrine utilization were found to be low among people with physical disabilities. Family support, latrine accessibility and past latrine modification were predictors of latrine utilization. Presence of entrance steps, lack of privacy, unavailability of handrails, lack of family support, narrow doors, distant location of latrine and elevated squatting foot rests were challenges mentioned by PPDs in consistently using latrines. Thus, responsible bodies should advocate for and design accessible and inclusive latrines, encourage family members to support people with physical disabilities in using latrines, and modify existing latrines to accommodate people with physical disabilities to improve the situation.

## Abbreviations

PPDs: People with physical disabilities, AOR, Adjusted Odds ratio
HH: Households
OR: Odds ratio
SPSS: Statistical Package for Social Sciences
WHO: World Health Organization
WASH: Water Sanitation and Hygiene
IGA: Income Generating Activity
